# Tone and word length across languages

**DOI:** 10.3389/fpsyg.2023.1128461

**Published:** 2023-06-22

**Authors:** Søren Wichmann

**Affiliations:** Cluster of Excellence ROOTS, Kiel University, Kiel, Germany

**Keywords:** tones, tonogenesis, word length, linguistic diversity, linguistic typology, language size

## Abstract

The aim of this paper is to show evidence of a statistical dependency of the presence of tones on word length. Other work has made it clear that there is a strong inverse correlation between population size and word length. Here it is additionally shown that word length is coupled with tonal distinctions, languages being more likely to have such distinctions when they exhibit shorter words. It is hypothesized that the chain of causation is such that population size influences word length, which, in turn, influences the presence and number of tonal distinctions.

## Introduction

Previous work has investigated factors that influence word length both across meanings on a subset of the Swadesh list and across languages (Wichmann and Holman, 2023). Across languages, a factor found to influence word length was population size. Aggregation across language families and six macroareas compared with similarly aggregated logs of population sizes showed an extremely strong (*r* = −0.92, *p* < 0.01) correlation. In other words, word length averaged across families and then across macroareas decreases as similarly averaged populations increase. This finding supports a suggestion in Wichmann et al. (2011, p. 193–194) of an existence of an inverse relationship between word length and population sizes, a suggestion which, in turn, followed an original proposal by Nettle (1995, 1998). The main insights from the study of Wichmann and Holman (2023) may be replicated from the basic data, which have been made available online at https://zenodo.org/record/6344024. Data on word length was based on averages across the 40 item word lists in ASJP (Wichmann et al., 2020), data which will also be used in the present study.

The present paper goes on to look at how presence/absence of tones as well as the number of tonal contrasts relate to mean word length. Languages with shorter words might be more susceptible to having tonal contrasts, and, beyond mere presence vs. absence, it seems worthwhile to test whether the number of tonal contrasts correlates with word length. For instance, SE Asia is famous for having a high concentration of tonal languages as well as for a tendency for languages to have monosyllabic words. In contrast, Australian languages tend to have long words and no tones. The aim of the work described in this paper is to test whether a relationship between word length and tones generalizes beyond such anecdotal cases. Research on ways that tonal distinction may emerge (tonogenesis), moreover, suggests a plausible causal connection between loss of segmental material and the gain of tonal contrasts. For instance, in an early stage of the development of Vietnamese, final /h/ and /ʔ/ can be assumed to have been preceded by phonetically falling and rising tonal intonational contours, respectively. Subsequently final /h/ and /ʔ/ were both lost, and the erstwhile phonetic prosodic difference on the preceding vowels turned into a phonological, tonal distinction (Haudricourt, 1954). Earlier (some time between 500BCE and 500CE), Chinese had undergone a similar development (Sagart, 1999). Such developments are not restricted to SE Asia. For instance, at least four languages of Mexico and Guatemala pertaining to different branches of the Mayan family have developed contrastive tones in the context of former laryngeals (Bennett, 2016, p. 497–498). Although it is far from all cases of tonogenesis that involve a loss of segmental material (cf. Michaud and Sands, 2020 for a recent review), documented cases of this particular pathway justifies the interpretation of an inverse correlation between tonal contrasts and word length as being non-spurious.

This paper seems to be the first to investigate the relationship between word length and tones across languages. Previously the relationship between word length and segment inventory sizes was examined, with somewhat ambiguous results. Nettle (1995) suggested the existence of an inverse relationship between word length and inventory sizes, but on a very small empirical basis. Moran and Blasi (2014, p. 234–236) and Wichmann and Holman (2023) brought more data to the table, also finding an inverse relationship, but the latter authors were not able to confirm a statistical significance of the findings. Further afield, Maddieson (2007) found positive correlations between the sizes of vowel and consonant inventories and the complexity of tonal systems, whereas syllable complexity and tone were negatively correlated according to his study.

## Materials

Conceivably, there are many options for obtaining information on word length and tonal distinctions across different languages. Potential sources for such information include textual corpora, dictionaries, grammars, and typological databases, where the last-mentioned type of source could possibly be constructed from any selection of the first three kinds of sources. The choices of sources of information for the present paper have been guided by two major criteria: comparability and coverage. Those criteria have led to the selection of large typological databases as sources of information. As in Wichmann and Holman (2023), the 40-item word lists in the lexical ASJP database (Wichmann et al., 2020) were chosen as a source of word length data because they represent around ¾ of the world’s languages, which makes for a better coverage than any other source. Additionally, the data are comparable since the words in the list pertain to one and the same fixed set of meanings and are transcribed phonemically in a standard way. As for the information on tone system, this comes from Phoible (Moran and McCloy, 2019) with some additions from the WALS chapter on tone (Maddieson, 2013) and The Database of Eurasian Phonological Inventories (Nikolaev, 2018). These sources together offer a coverage of around ¼ of the world’s languages and consistency in the type of data targeted, namely phonological systems. Although the description of phonemic distinctions may vary between researchers (Moran, 2012), the counts of tonal distinctions are at least similar in the sense that they aim to include all distinctions attested in a given language (as opposed to, say, all distinctions attested in some corpus).

One criterion that might be considered in addition to coverage and comparability is representativeness. The average length of items pertaining to a short word list is not necessarily representative of the lexicon as a whole or mean word length in usage. Nor is a number of tonal distinctions necessarily representative of actual usage, since two languages might each make use of the same number of distinctions but with widely different distributions of frequencies. There are, however, two major reasons why the criterion of representativeness is not given priority here. First, representativeness is not a trivial notion, but one that requires potentially controversial assumptions concerning the entity represented. If a language is considered to be the sum of all discourses produced using a certain code, then a representative sample would be a large corpus covering different genres and modalities. If a language is considered to be a set of lexical and phonological elements combined through some syntagmatic rules, then a representative sample might be a selection of lexical elements, perhaps subjected to selected syntagmatic operations. Thus, it is not clear how to even define a criterion of representativeness. Another major reason why representativeness is not given priority is that it will often clash both with the criterion of comparability, which is a principle that cannot be relinquished, as well as with the criterion of coverage, which is more flexible than comparability, but also important. For instance, among the many corpora existing for various languages, most would not be comparable since they would be different in contents, treating different topics and representing different genres, as well as in form, being encoded in different orthographies. Moreover, for many languages no corpora are available at all, compromising the criterion of coverage.

The optimal sample is neither easy to define nor easy to obtain. Therefore it would be a relief to be able to show that various sources of word length data actually produce similar results. In the following I will report on some analyses indicating the degree to which this wish may be fulfilled. Briefly, I compare counts of word length based on ASJP 40-item lists with (1) 100-item lists from ASJP, (2) 985-item lists from NorthEuralex (Dellert et al., 2020), and (3) corpora from TeDDi (Moran et al., 2022) representing (3a) Bible texts, and (3b) versions of the Universal Declaration of Human Rights. The reader who wishes to skip the details may jump to Table 1 where the results are gathered.

**Table 1:** Correlations (Pearson’s *r*) between mean word length of 40-item ASJP word lists and other data sources (in all cases *p* < 0.001).

Data source	*r*	*N*
100-item ASJP lists	0.94	1250
985-item NorthEuraLex lists in ASJPcode	0.78	105
985-item NorthEuraLex lists in original orthography	0.68	92
Universal Declaration of Human Rights	0.58	36
Bible texts	0.60	49

Before describing the comparisons with other sources of word length data, let me present the data actually used. For the present purposes a word is defined as the typical source of an ASJP item, which is an entry in a dictionary marked as a single, separate string by leading and trailing spaces and providing a translational equivalent of a specific concept commonly lexicalized throughout the languages of the world. Mean word length of a language is defined as the mean across such ASJP items. If two synonyms are given for a certain concept, an average length is used here, and if more than two synonyms are given, only the two first ones listed are taken into account. Phrases (anything with one or more spaces in it) are ignored. All identifiable inflectional affixes were removed during the transcription of ASJP items, so in many cases ‘stem’ might actually be a more adequate description of the contents of the ASJP database, although the vast majority of the entries would be words in a normal sense. These words (or word proxies) are transcribed using ASJPcode (Brown et al., 2013), a transcription system which merges phonemes into classes of phonemes but adequately represents the number of phonemes in words. It operates with 34 consonant and 7 vowels symbols, a nasalization symbol, and modifiers indicating that sequences of two or three symbols are to be interpreted as single phonemes. Additionally, there is a symbol (%) to indicate that a word is a borrowing (this is not systematically applied). For each language as defined by ISO 639-3, the word length of a certain item on the 40-item list is averaged across the word lists pertaining to one and the same ISO 639-3 language, in case more than one is available (on average there is close to two word lists per language). The following list represents the doculect english. It is not necessarily a typical list, but it is one that any reader can immediately relate to (for other examples, the reader may visit https://asjp.clld.org/languages). The total count of phonemes in this list is 134, which, divided by the list length of 40, yields an average word length of 3.35.

Ei ‘I,’ yu ‘you,’ wi ‘we,’ w3n ‘one,’ tu ‘two,’ %prs3n ‘person,’ fiS ‘fish,’ dag ‘dog,’ laus ‘louse,’ tri ‘tree,’ lif ‘leaf,’ %skin ‘skin,’ bl3d ‘blood,’ bon ‘bone,’ horn ‘horn,’ ir ‘ear,’ Ei ‘eye,’ noz ‘nose,’ tu8 ‘tooth,’ t3N ‘tongue,’ ni ‘knee,’ hEnd ‘hand,’ brEst ‘breast,’ liv3r ‘liver,’ driNk ‘drink,’ si ‘see,’ hir ‘hear,’ dEi ‘die,’ k3m ‘come,’ s3n ‘sun,’ star ‘star,’ wat3r ‘water,’ ston ‘stone,’ fEir ‘fire,’ pE8 ‘path,’ %maunt3n ‘mountain,’ nEit ‘night,’ ful ‘full,’ nu ‘new,’ nem ‘name.’

The word length data used in the analyses of this paper is drawn from a file called Data-01 ASJP data raw.txt, available at https://zenodo.org/record/6344024. The file was previously used in Wichmann and Holman (2023). It contains columns for ISO 639-3 codes, doculect names, language codes and family classifications from WALS (Dryer and Haspelmath, 2013) and Glottolog (Hammarström et al., 2021), coordinates, population figures from Ethnologue (Simons and Fennig, 2017), word length averaged over the 40 ASJP items and over the entire 100-item Swadesh list when available; there are also assignments of ‘area,’ ‘continent,’ and ‘macrocontinent’ from Autotyp (Bickel et al., 2017), as well as some other columns of less relevance in the present context. Word length data can be obtained from ASJP for 5289 languages (here and henceforth as defined by ISO 639-3).

In order to estimate the extent to which word length data based on the 40 ASJP items compares to some other sources of word length data I drew samples from the following sources: 100-item lists that are also part of the ASJP database, longer word lists in NorthEuraLex (Dellert et al., 2020) and text corpora from TeDDi (Moran et al., 2022). These comparanda are meant to represent samples that may be conceived of as being more representative of the involved languages than the 40 ASJP items. Mean word length for 100-item word lists are directly obtained from the same dataset used here for the 40-item lists. NorthEuraLex contains 1016-item word lists for 107 Eurasian language varieties in transcriptions that include standard orthographies and, conveniently, also ASJPcode. In order to enhance comparability I removed the least attested items (31 items attested in less than 98 languages). I also removed two languages that had been excluded from the ASJP data for not being anyone’s current mother tongue, namely Latin and Standard Arabic. For the remaining 105 985-item word lists average word lengths were computed from the ASJPcode transcriptions. Additionally, for 92 languages associated with alphabetical writing systems, word length was computed from orthographical forms. As examples of text corpora I extracted Universal Declaration of Human Rights texts and Bible texts from TeDDi. TeDDi is conceived of as a sort of complement to WALS (Dryer and Haspelmath, 2013), containing corpora for 89 languages that belong to the core WALS sample of 100 languages.^1^ While the corpora are generally heterogeneous, Bible texts and Universal Declaration of Human Rights texts recur among them. Only languages represented in alphabetical writing systems could be used. Left were 36 languages with Universal Declaration of Human Rights texts and 49 languages with Bible texts from which to extract mean word lengths. Since TeDDi has a good areal and genealogical spread of languages and offers the corpora nicely organized in a single R object it is a convenient choice of sources. It goes without saying that larger sets of corpora could have been used, but for the present purposes this would seem unnecessary.

Results of comparing word length counts across languages for the different sources are displayed in Table 1. When increasing the representativeness of the word lists from 40 to 100 and then to 985 items the correlation changes from 1.00 to 0.94 and then to 0.78. From the point of view of the presumably more representative sample this can be interpreted as an increase in adequacy, first by 0.06 (1.00–0.94) when going from 40 to 100 items and then an additional 0.16 (0.94–0.78) when going from 100 to 985 items. Continuing down the table we observe a difference of 0.10 correlation between the ASJPcode and original orthographical NorthEuraLex word lists. In this case the difference can only be interpreted as a loss, because the systematic ASJPcode should make for better comparability than traditional orthographic forms. When moving to the corpora, we observe a correlation of ~0.6. Because of the two different versions of transcriptions contained in NorthEuraLex we expect that a systematic phonemic transcription of a corpus would have yielded an around ~0.1 better correlation with the 40-item ASJP lists, i.e., the correlation with corpora would then be ~0.7.

As discussed above, representativeness is not a straightforward and uncontroversial notion. Still, we might consider either more extensive word lists or corpora as more representative of a language than the 40 ASJP items. Results using short word lists would be more different from results using corpora than from results using long word lists, but in either case the results would not be radically different if we were able to obtain systematic, phonemic transcriptions for the long word lists or the corpora. Such transcriptions, however, are rarely available, compounding the general lack of availability for long word lists and corpora. Thus, to conclude these experiments regarding alternative data sources: alternative data sources might be preferable from the point of view of representativeness, but for many practical purposes they would be problematical because of the challenges incurred by limitations on availability and the existence of different orthographical systems. Moreover, the relatively high correlations found between 40-item ASJP lists and the other data sources suggest that the short word lists can reasonably be used as a proxy for those other kinds of more extensive sources.

Data on the number of tonal distinctions can be obtained from Phoible (Moran and McCloy, 2019), with a few modifications. Phoible includes data from The Database of Eurasian Phonological Inventories (Nikolaev, 2018, henceforth EURPhon), but the data on tones were not included. Instead, all languages from EURPhon are represented as not having tones. Therefore, the EURPhon data in Phoible were removed and replaced by data coming directly from EURPhon. Moreover, a few errors were spotted relating to language supposedly not having tones in the Phoible “PH” dataset.^2^ Since a ‘0’ seems to sometimes means ‘not applicable’ rather than absence of tones, all data points pertaining to the PH dataset encoding a language with 0 for tones were removed. Data from another 257 languages can be added from the WALS chapter on tone (Maddieson, 2013), extending the data available on the simple presence or absence of tones. After excluding languages not suitable for the present research (artificial, creoles, pidgins, fake, speech registers, unclassified, mixed languages, languages for which less than 20 out of the 40 items are attested) and extracting the data overlapping between ASJP and the sources for tonal data, 1,380 languages remain. That is, for 1,380 languages both word length counts and counts of tonal distinctions are available. For an additional 108 languages there was data on presence vs. absence of tones, but not the number of tones (beyond 0). Just as for the word length counts, the unit of analysis is a language as defined by ISO 639-3. Therefore, in case more than one inventory is available for an ISO 639-3 language, the number of tones is averaged.

Finding good alternatives to such data on tonal distinctions coming from typological databases seems even less viable than the alternatives to word length data that we discussed. Plausibly it might be an advantage if data on tonal distinctions came directly from the same sample of words from which word length counts are produced, for instance. But many of the sources of lexical data used do not adequately record tones, and even for those that do, the ASJP database does not include this information.

## Methods

R scripts (R Core Team, 2022) for processing the data from ASJP, Phoible, and WALS and for performing analyses is available online (see the Data Availability Statement). The relationship between tones and word length is explored in a variety of ways. A linear mixed effects model was fitted using the lme4 package (Bates et al., 2015). The lme4 package is again involved in a logistic regression analysis. These analyses mainly served to generalize across language families. Various aspects of data preparation and plotting involved the dplyr (Wickham et al., 2023), tibble (Müller and Wickham, 2022), ggplot2 (Wickham, 2016), rworldmap (South, 2011), and colorspace (Zeileis et al., 2020) packages.

In order to investigate whether a negative correlation between word length and the number of tonal distinctions also shows up within families I carried out linear regression and phylogenetic correlation. The sign and magnitude of the linear regression provides information on the general nature of the relationship. Non-independence of the data, however, render *p*-values non-trustworthy. Instead, the phylogenetic correlation analysis (Pagel, 1994, 1997, 1999) serves to estimate the likelihood of a model where the word length and the number of tonal distinctions are assumed to be correlated. This analysis required special efforts because some components of the pipeline were not available and had to be developed. The idea of the analysis is to map the word length and tone data onto phylogenetic trees having distinctive branch length in order to see whether the evolutions of the two features are coupled. In order to achieve this, I used trees from Glottolog (Hammarström et al., 2021) pruned such that only those languages appear for which lexical distances could be computed and for which data on tones and word length were available. The Glottolog trees were then supplied with branch lengths based on lexical distances from ASJP, and the phylogenetic correlation analysis could be carried out using BayesTraits (Pagel et al., 2004).

Continuing with more detail on the pipeline for correlated evolution, the first step was to compute lexical distances in order to be able to supply branch lengths. In a formally similar kind of analysis of correlated evolution involving some linguistic traits, Shcherbakova et al. (2022) used the ASJP-based global tree of Jäger (2018) as well as a few Bayesian trees from the literature representing larger language families. The alternative of using Glottolog trees with added branch lengths ensures a degree of consensus regarding the structure of the tree as well as transparency and consistency; it avoids the awkward notion of a single world language family; and it allows for using the latest updates of ASJP (here version 20 is used; Jäger’s tree is based on version 17). The lexical distances represent averages of a length-normalized Levenshtein distances (edit distances) across word pairs on the 40-item ASJP word lists: for each pair of words referring to the same concept the Levenshtein distance is found. (A convenient function for this is the adist() function of Base R). It is normalized by the length of the longest of the two strings compared. In various papers since Holman et al. (2008) this has been referred to as LDN (‘Levenshtein Distance Normalized’). Wichmann et al. (2010a) showed empirically that a further modified version of the Levenshtein distance (called LDND for ‘Levenshtein Distance Normalized Divided’) is better for comparisons potentially involving unrelated languages, but since we are here only comparing related languages the less computationally intensive LDN distance suffices. It has been implemented in the interactive software of Wichmann (2023). This has many ways of selecting doculects and various choices of analyses and output. For the present purposes I exclude proto-languages, ancient attested languages, languages gone extinct between ancient times and around 1700; I choose only one doculect per ISO 639-3 language, namely the one represented by the longest word list; and I restrict word lists to those that have at least 20 items. The program operates through menus asking for input from the user. For instance, in order to produce an LDN matrix for Nilotic in an output file called Nilotic_LDN.txt the user input would supply the following 15 responses when the program is first used (using spaces to separate responses): 2 1 2 1 2 1 2 Nilotic 1238 m 20 1 3 a 2 Nilotic_LDN.txt. For convenience, the relevant output matrices are supplied online (see Data Availability Statement).

Continuing with more detail on the pipeline for correlated evolution, adding lexical distances from ASJP to Glottolog trees requires a matching of ASJP doculect names and Glottocodes. This is mainly achieved using the file languages.csv from https://zenodo.org/record/7079637, with some modifications of matches: in cases where an ASJP doculect is matched with a glottocode representing the ‘dialect’ or ‘family’ level, the phylogenetically closest ‘language’-level glottocode is assigned instead. This procedure makes sense conceptually and is also required technically because later in the pipeline the keep_as_tip() function of the glottoTrees package (Round, 2021) will be used for tree pruning, and this function will stop and issue an error message if the result of pruning a tree would leave a taxon as a descendant of another taxon. For instance, standard_albanian is assigned to the glottocode alba1267, which is a ‘family’-level label belonging to a higher taxonomic level than, for instance, albanian_tosk (tosk1239). In fact, the two doculects should both be assigned to tosk1239, since the Tosk dialect is the basis for the standard language. More commonly, however, the problem is that a doculect is assigned to the ‘dialect’ level. For instance, bosnian is assigned to ‘Bosnian standard’ (bosn1245), which itself is a ‘dialect’ of ‘Eastern Herzegovinian Shtokavian’ (east2821), which itself is a ‘dialect’ of ‘New Shtokavian’ (news1236), which itself is a ‘dialect’ of ‘Shtokavski’ (shto1241), which itself is a ‘dialect’ of the ‘Serbian-Croatian-Bosnian’ (sout1528) ‘language.’ While this is the only case encountered of as many as four levels of ‘dialect’ it receives the same treatment as less complicated cases, namely a direct reassignment of the dialect to the language level (in this case changing bosn1245 to sout1528).

After having prepared distance matrices for those ASJP languages for which information on tonal distinctions are available and having assigned glottocodes to them, the Glottolog trees are pruned so as to only contain the languages also appearing in the distance matrices. This is done using the keep_as_tip() function of glottoTrees (version 0.1; Round, 2021). While this works smoothly once the problems mentioned in the previous paragraphs are taken care of, its output needs further processing in case internal non-branching nodes are retained after pruning. For instance, let two final taxa (tips) A and B be united under an internal node Int. In the Newick notion^3^ such a tree would be represented as ((A,B)int,C). If B is removed during the pruning process the function will still leave Int within the tree, even if this node is not branching, in Newick notion: ((A)int,C). Such ‘phantom’ nodes are not tolerated by nnls.tree() of Phangorn 2.10.0 (Schliep, 2011), the function used here to supply tree with distinctive branch lengths. Indeed, they are generally not foreseen by phylogenetic software. For instance, MEGA (Tamura et al., 2021) will not be able to display a tree with non-branching nodes. Fortunately, there is a simple solution to this problem. Since the internal nodes and placeholder branch lengths of 1 of the Glottolog trees are not needed, these features can be removed using regular expressions. This will leave only tip labels and brackets, easing further edits to the Newick format. A non-branching node will appear as a set of ‘phantom’ brackets not containing commas not already contained in other brackets contained within the ‘phantom’ brackets. In our simplest-possible example there would be a set of ‘phantom’ brackets left around A as it is deprived of its sister B: ((A,B)int,C) → ((A,B),C) → ((A),C). In cases where the pruned taxon is not the terminal sister of a single other taxon, some further look-around is required to find the two friends making up a pair of ‘phantom’ brackets, as in the case of (((A,B)),(C,D)) ← (((A,B),E),(C,D)), where the culprits are the extra brackets around (A,B). These cases will be identifiable as two consecutive opening brackets that are members of a set of brackets which includes closing brackets which are likewise consecutive. Based on these insights, an algorithm was implemented in my fix.non.br() function in the phylogenetic_correlation.R script supplied along with this paper. Other functions, from various packages, that were used in the tree manipulation procedures included read.tree(), write.tree(), drop.tip(), and write.nexus() from ape 5.7 (Paradis and Schliep, 2019); and str_split() and str_sub() from stringr 1.5.0 (Wickham, 2022).

At this point in the pipeline a distance matrix and a Newick tree is available for each language family (where a family is required to have 6 or more members). This is the input needed for Phangorn’s nnls.tree() function, which is used for supplying the Glottolog trees with branch lengths. Previously Dediu (2018) similarly used this function to supply branch lengths from various sources to language family trees of different extractions (Ethnologue, WALS, Autotyp, Glottolog), and I am inspired by this work but use my own implementation of the process. What nnls.tree() does, summarily stated, is to estimate branch lengths such that patristic distances among taxa, i.e., the distances between taxa along the tree, best approximate the distances in the supplied matrix. This is done by applying the least squares criterion, minimizing the sum of squared errors. A blog post by Revell (2011) provides an entry point for better comprehension. It is of interest to look at how well the resulting patristic distances fit the original LDN distances. This is done for each family using the mantel.rtest() of ade4 1.7.19 (Dray and Dufour, 2007). The resulting *r* values, which are all significant at the *p* < 0.01 level, are reported in Table 2, in descending order. I am not aware of similar tests of other, comparable branch length fitting outcomes, so it is difficult to know what to require from the results, but the fits certainly seem good enough to at least pass a sanity test: the results are approximately normally distributed around a high mean of 0.93.

**Table 2:** Results of mantel tests for LDN and patristic distances in trees supplied with branch lengths.

Family	*r*	Family	*r*
Otomanguean	0.981	Austronesian	0.938
Central Sudanic	0.972	Nuclear Trans New Guinea	0.928
Tai-Kadai	0.970	Indo-European	0.928
Mande	0.967	Afro-Asiatic	0.928
Kadugli-Krongo	0.960	Sino-Tibetan	0.923
Nilotic	0.955	Atlantic-Congo	0.899
Athabaskan-Eyak-Tlingit	0.944	Ta-Ne-Omotic	0.810
Austroasiatic	0.939	Salishan	0.772

As the last element of the correlated evolution pipeline the software BayesTraits in its most recent instantiation, version 4.0.1 (Meade and Pagel, 2023), is put to work. Similarly to Shcherbakova et al. (2022), I follow the recommendations of the BayesTraits manual for testing correlations between continuous traits (Meade and Pagel, 2023, p. 37–38). The assumption here is that traits evolve as random walks. To estimate whether two traits are coevolving, a complex model assuming a correlation is compared with a simple model in which the correlation is set to zero. The strength of the complex model over the simple one is estimated through a log Bayes Factor, calculated as 2 * (log marginal likelihood complex model – log marginal likelihood simple model). These log Bayes Factors may be interpreted as in Table 3, following Raftery (1996).

**Table 3:** Interpretations of log Bayes Factors (from Raftery, 1996, p. 165).

Log Bayes Factors	Evidence for alternative hypothesis
<0	Negative (supports null hypothesis)
0–2	Barely worth mentioning
2–5	Positive
5–10	Strong
>10	Very strong

## Results

**Figure 1:**
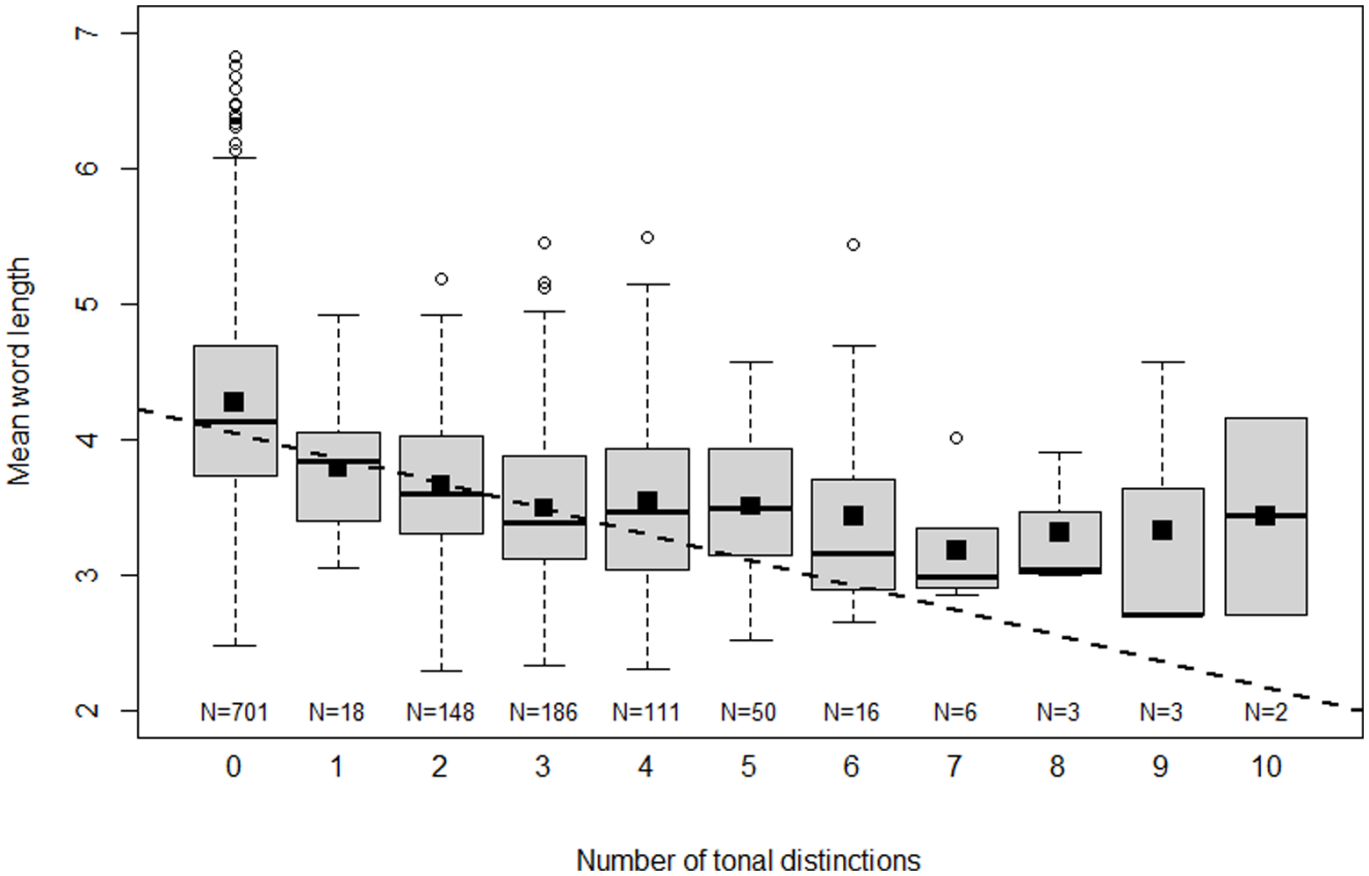
Boxplots of mean word length for different numbers of tonal distinctions. Small black squares represent means and the dashed line is a linear fit of all raw values of mean word length and the number of tonal distinctions.

We begin to explore the nature of the relationship between word length and the number of tonal distinctions by means of the boxplots in Figure 1. Each boxplot represents mean word length values for a certain number of tonal distinctions. Sometimes, when more than one language variety is involved, the number of tonal distinctions of an ISO 639-3 language (the unit of analysis) is not a whole number. For the purpose of the graph, the number has then been rounded off to the nearest integer. Small squares represent means. The fitted line is not based on any kind of binning but represents the linear fit of all values of number of tonal distinctions and mean word length. Although this fit over the entire range is decent (*R*^2^ = 0.196), the graph suggests that the correlation mainly holds for values of tonal distinctions from 0 to 3, while the relationship for values in the range 4–10 is at best weak. Apparently there is a lower limit on vowel length of 2–3 segments that languages cannot cross without losing too much in terms of expressive means. But once this limit is reached, tonal systems can still develop in complexity for reasons other than through compensation for segment loss. Referring to three or more tones as ‘several,’ we can say that mean word length is a strong predictor of whether a language will have zero, one, two or several tones. The number of tones above three, however, would seem not to depend appreciably on this factor, at least as far as we can judge from the available data, which is relatively limited for the complex systems. Still, in order to avoid manufacturing of results, we do not combine three or more tones in one bin, but continue to operate with the original range of values in subsequent analyses.

Before exploring the relationship between the number of tonal contrasts and mean word length further, we ask whether the relationship is statistically significant in the first place. The question is answered by formulating a linear mixed effects model with the number of tonal contrasts as a function of mean word length (predictor variable) and random effects represented by Glottolog family membership and membership of one of the following ‘continents’ of Autotyp: Africa, Western and Southwestern Eurasia, North-Central Asia, South and Southeast Asia, New Guinea and Oceania, Australia, Eastern North America, Western North America, Central America, and South America (when a family is spread over more than one continent all members are assigned to just one continent, namely the one from which scholars would normally assume the family to have originated, cf. discussion of received views in Wichmann et al., 2010b; a list of the decisions taken is in the script tones.R, provided online). When trying to estimate both slopes and intercepts for the random effects singular fits arose, so here only the intercepts are estimated. The summary of the model is found in Box 1.

BOX 1. **Summary of linear mixed effects model with number of tonal contrasts as a function of mean word length (predictor) and family & continent (random effects).**

 Linear mixed model fit by maximum likelihood [‘lmerMod’]

 Formula: count_tones ~ forty_mean + (1 | continent) + (1 | glot_fam)

    Data: pho2

     AIC    BIC   logLik   deviance    df.resid

     4781.9  4808.0  -2385.9    4771.9    1375

    Scaled residuals:

     Min    1Q   Median   3Q   Max

   -2.4076   -0.3701  -0.0644  0.2672  5.8136

 Random effects:

   Groups   Name   Variance  Std.Dev.

   glot_fam  (Intercept)  0.4780  0.6914

   continent  (Intercept)  0.2387  0.4885

   Residual        1.6970  1.3027

 Number of obs: 1380, groups: glot_fam, 178; continent, 10

 Fixed effects:

         Estimate Std.  Error  t value

 (Intercept)    3.12390    0.34192  9.136

 forty_mean   −0.56280    0.06764   −8.320

Of perhaps most interest in this output is the coefficient −0.563, which shows that around half a tonal distinction is gained per one segment decrease of word length.

Using the anova() function, the full model as fitted by lmer() is compared to a reduced model where the number of tonal distinctions is a function of its own mean, with the random effects retained. The output of this comparison shows the difference between the models to be highly significant (*Χ*^2^(1) = 66.32, *p* < 0.0001), and the smaller AIC and BIC values and higher log likelihood of the full model also indicate the importance of mean word length as a predictor of the number of tonal contrasts (Box 2).

BOX 2. **Summary of comparison of full model (cf. Box 1) with a reduced model where the number of tonal contrasts is removed as predictor variable.**

 reduced_model: count_tones ~ 1 + (1 | continent) + (1 | glot_fam)

 full_model: count_tones ~ forty_mean + (1 | continent) + (1 | glot_fam)

          npar   AIC   BIC   logLik deviance  Chisq  Df  Pr(>Chisq)

 reduced_model    4  4846.2  4867.1  -2419.1  4838.2

 full_model      5  4781.9  4808.0  -2385.9  4771.9  66.315  1  3.843e-16  ***

**Figure 2:**
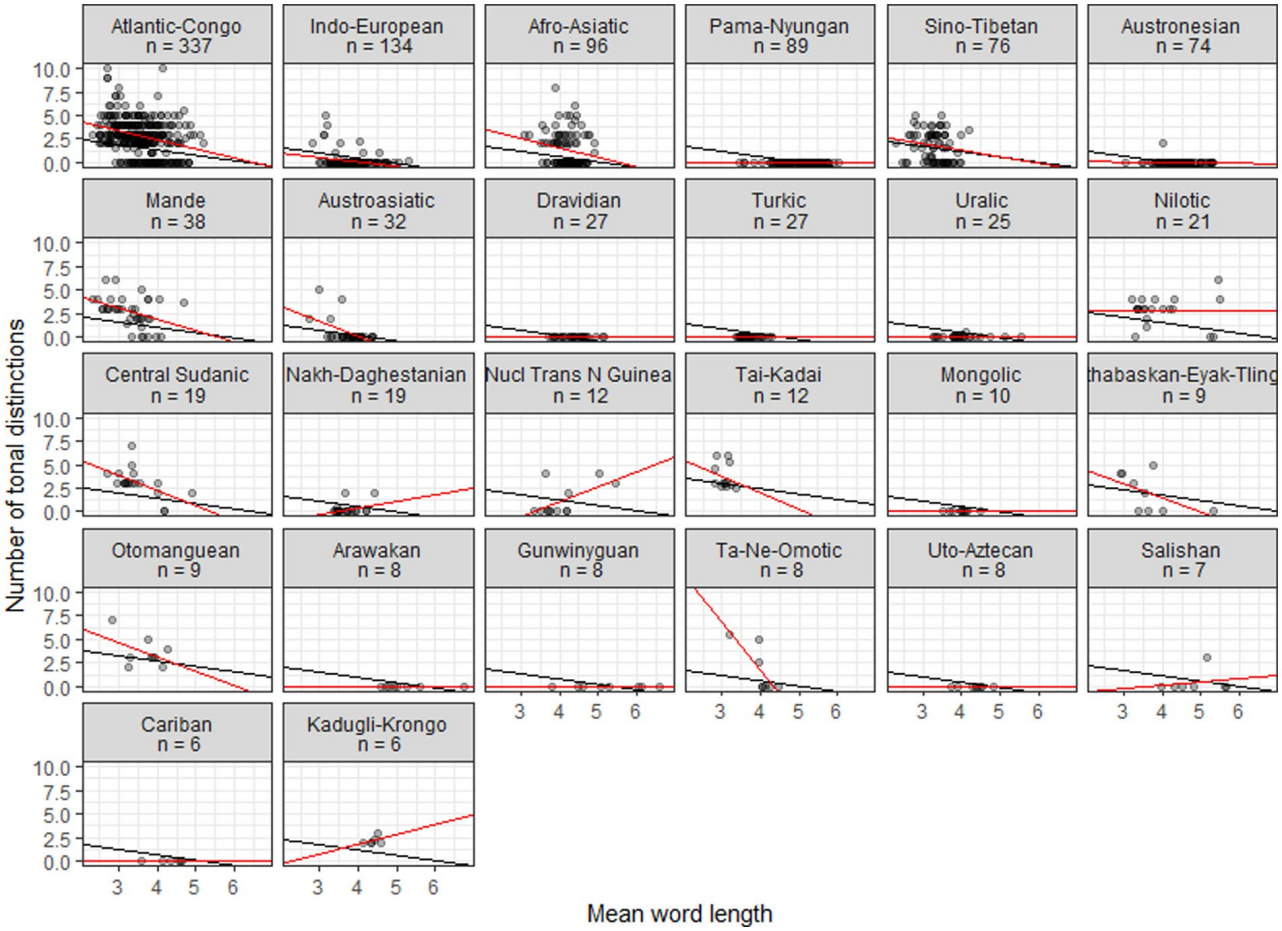
Scatterplots of tonal distinctions as a function of mean word length in families with six or more members. Black lines show fits to a general mixed linear model, with intercepts varied; red lines show fits to individual linear models.

**Figure 3:**
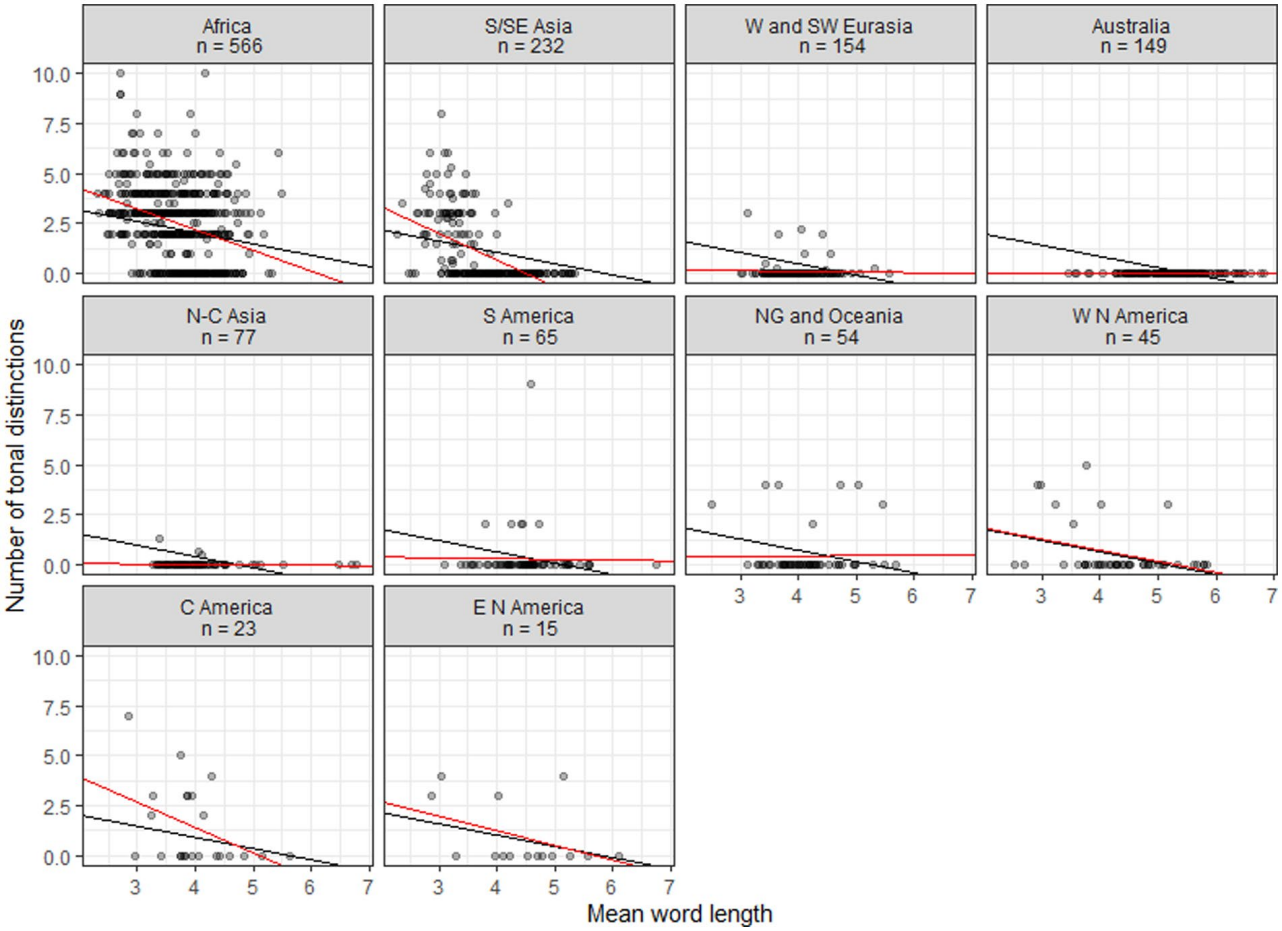
Scatterplots of tonal distinctions as a function of mean word length in continents. Black lines show fits to a general mixed linear model, with intercepts varied; red lines show fits to individual linear models.

Figures 2, 3 plot the data for, respectively, families with six or more members and continents. Black lines show the linear regressions produced by the mixed model, where only intercepts are varied. Red lines show linear regressions based on the data for individual families or continents. Typically there is a relatively good agreement between the fits of the general linear model and individual linear models for areas and larger families where tonal languages abound, while poorer fits emerge for areas and families where tonal languages are uncommon or absent; for small families some fits are probably in disagreement mainly because of small sample sizes. For continents there are similarly good agreements whenever tonal languages are common.

The family scatterplots with regression lines that tend to show negative slopes in Figure 2 strongly suggest that once tones are more than sporadically present in a family they will have developed in tandem with decreased word length. Fitting a linear model, however, ignores the diachronic perspective—it treats the languages as a pile of fallen leaves having no identifiable connection to specific branches in the tree that they come from. This represents a huge loss of information. In order to estimate the likelihood of a model where the developments of word length and tones are coupled, we need to include the tree structure connecting the languages in the analysis, making use of comparative methods from biology (Harvey and Pagel, 1991). Specifically, we use tree topologies from Glottolog (Hammarström et al., 2021), pruned such as to contain only the languages of interest and supplied with distinctive branch lengths based on lexical distances (normalized Levenshtein distances or LDN) calculated from ASJP data (Wichmann et al., 2022). Subsequently we feed the trees and the data on word length and tonal distinctions to BayesTraits (Meade and Pagel, 2023). The results, again reporting on families with six or more members, are in Table 4. This shows the log Bayes Factors, which express the amount of support for a model of correlated evolution and which may be interpreted following the guidelines in Table 3. Table 4 also shows Pearson’s *r* for the (non-phylogenetic) correlations between tones and word length (cf. the red fitted lines in Figure 2), mainly in order to remind us of the sign of the correlation.

**Table 4:** Log Bayes factors for phylogenetic correlation of tone and word length, Pearson’s *r* for conventional correlations of the same variables, and the number of languages.

Family	LogBF	*r*	*N*
Austroasiatic	9.43	−0.525	32
Atlantic-Congo	8.13	−0.297	337
Afro-Asiatic	5.93	−0.196	96
Ta-Ne-Omotic	5.69	−0.774	8
Indo-European	4.69	−0.277	134
Nuclear Trans New Guinea	4.03	0.594	12
Central Sudanic	2.79	−0.557	19
Athabaskan-Eyak-Tlingit	2.22	−0.526	9
Otomanguean	2.03	−0.444	9
Salishan	0.78	0.197	7
Sino-Tibetan	0.76	−0.166	76
Mande	0.67	−0.399	38
Austronesian	0.54	−0.099	74
Nilotic	0.53	0.004	21
Kadugli-Krongo	−0.24	0.432	6
Tai-Kadai	−0.49	−0.218	12

What emerges from Table 4 is that correlated evolution of tone and word length is supported to various degrees (LogBF >2) in 9 cases. Another 5 cases are ‘not worth talking about’ and only 2 cases (Kadugli-Krongo, Tai-Kadai) support the null hypothesis. The conventional correlation analysis indicates a negative relationship in 12 cases and a positive relationship in 4 cases. Among the latter cases, however, only Nuclear Trans New Guinea (nTNG) finds support from the phylogenetic correlation. When looking more closely at the data it turns out that only 4 out of the 12 nTNG languages are tonal. Moreover, nTNG is a contested family (Wichmann, 2013). If tones are only attested in a few languages and if the genealogical relationships are uncertain we have reasons to discount these results. The Tai-Kadai languages all have 2.5–6 tones and word lengths of 2.83–3.35. Thus, they belong to the range of the distribution of word length and tone where the relationship breaks down, presumably because a floor on the word length has been reached (cf. Figure 1).

Another way of assessing the importance of mean word length for tones is to look at the mere presence vs. absence of tones and infer the probability of having tones as a function of mean word length. We perform this analysis using the glmer() function of the lme4 package. Presence/absence, represented by the digits 1 and 0, is fitted to the same model as earlier, with mean word length as predictor and continent and area as random effects (formulaically: p_a ~ forty_mean + (1 | continent + (1 | glot_fam), data = pho3, family = binomial). The summary of the model is found in Box 3.

BOX 3. **Summary of generalized linear mixed model with presence/absence of tone as a function of mean word length (predictor) and family & continent (random effects).**

   Generalized linear mixed model fit by maximum likelihood (Laplace Approximation) [‘glmerMod’]

 Family: binomial ( logit )

   Formula: p_a ~ forty_mean + (1 | continent) + (1 | glot_fam)

    Data: pho2

     AIC  BIC  logLik  deviance  df.resid

   1209.8  1231.0  −600.9  1201.8   1484

   Scaled residuals:

    Min  1Q  Median  3Q  Max

 -3.8615  -0.3149  −0.0719  0.4266  8.6409

   Random effects: Groups  Name  Variance Std.Dev.

    glot_fam (Intercept)   2.20  1.483

    continent (Intercept)   2.34  1.530

   Number of obs: 1488, groups: glot_fam, 201; continent, 10

   Fixed effects:     Estimate  Std. Error z value Pr(>|z|)

   (Intercept)  3.0598642 0.0007789 3929 <2e-16 ***

   forty_mean −1.1157857 0.0007796 −1431 <2e-16 ***

Just as done for the model with the count_tones predictor, the full model with the p_a (presence/absence) predictor is compared to its counterpart without this predictor through anova(). Again we find strong support (*Χ*^2^(1) = 50.49, *p* < 0.0001, smaller AIC and BIC, higher log likelihood) for the full model (Box 4).

BOX 4. **Summary of comparison of full model (cf. Box 3) with a reduced model where presence/absence of tone is removed as predictor variable.**

   reduced_binary_model: p_a ~ 1 + (1 | continent) + (1 | glot_fam)

   binary_model: p_a ~ forty_mean + (1 | continent) + (1 | glot_fam)

             npar   AIC   BIC   logLik   deviance  Chisq  Df  Pr(>Chisq)

   reduced_binary_model   3  1258.3  1274.2  -626.15  1252.3

      binary_model    4  1209.8  1231.0  -600.90  1201.8  50.491  1  1.197e-12 ***

**Figure 4:**
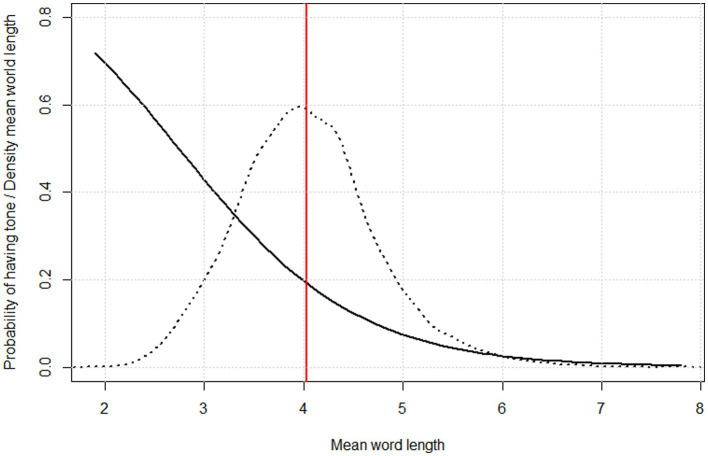
Probability of having tone as a function of mean word length, as inferred through logistic regression (solid curve) overlaid on a density plot of mean word length distribution across 5044 languages in ASJP (dotted curve) and showing the overall mean of mean word lengths (red vertical line).

The intercept and slope are now retrieved from the summary of the model and we can infer probabilities for different values of mean word length using the plogis() function of base R’s stats component. Results are shown in Figure 4. Here the curve is overlaid on a density plot of raw word length data in all the 5044 languages from ASJP available for this study. Figure 4 shows that the probability of having tones decreases as mean word length increases from the minimum (1.93 segments) to the maximum (7.73 segments).

**Figure 5:**
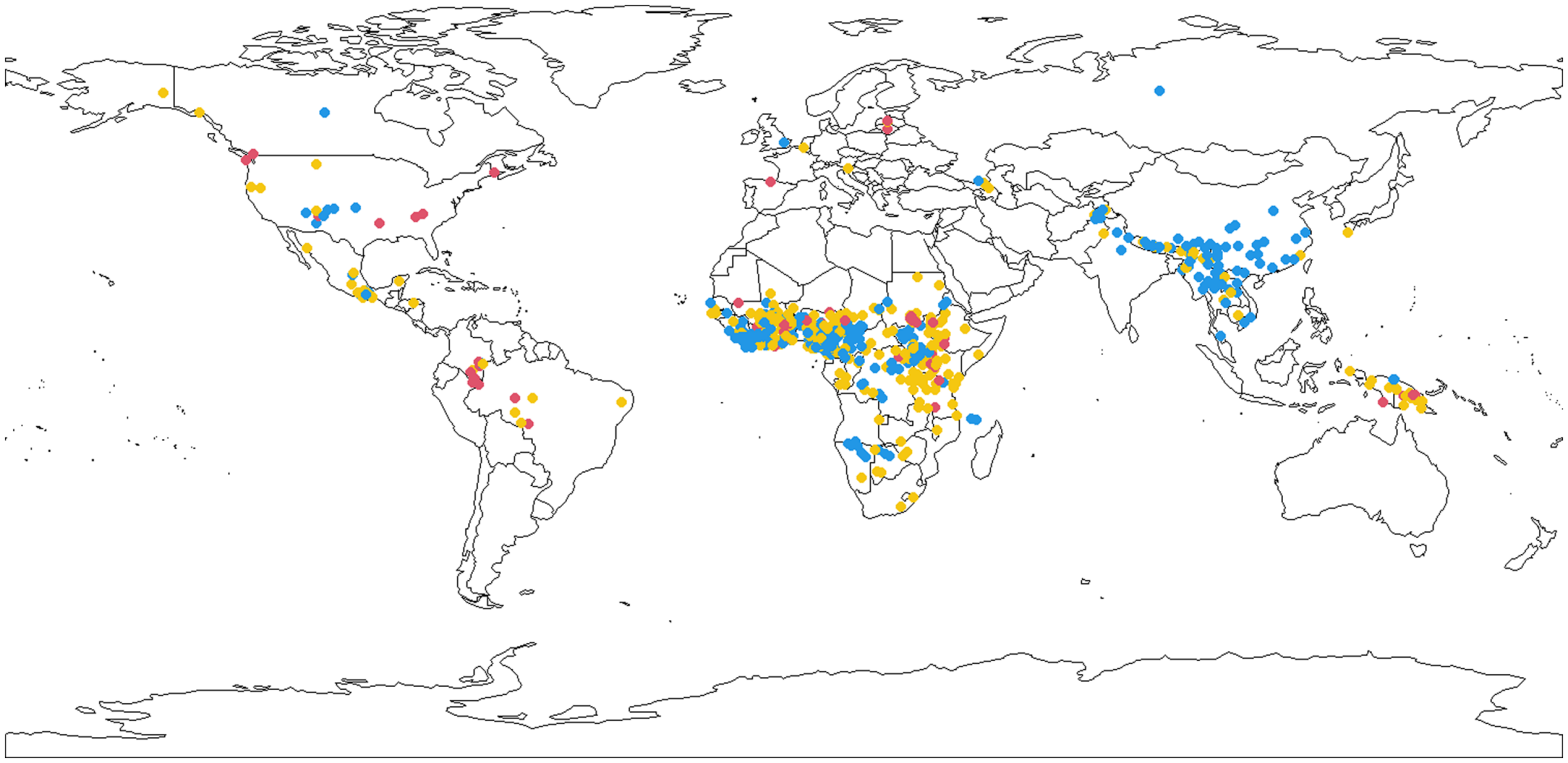
A map of tonal language with short (blue), intermediate (yellow), and long words (red).

As is well known from other surveys, including the WALS chapter on tones by Maddieson (2013), the main concentrations of tonal languages are in Subsaharan Africa and SE Asia. Figure 5 adds information on word length to the information on the presence of tonal languages. For the purposes of this map the tonal languages in our dataset were divided into three categories according to the quartiles of mean word length to which they belong: languages with short words (1st quartile, colored blue), languages with long words (4th quartile, colored red), and languages with intermediate word length values (2nd and 3rd quartiles, colored yellow). The map reveals that associations between tones and long words tend to be proportionally more common outside of the core tonal areas (Subsaharan Africa, SE Asia) than inside them. Most strikingly, in South America and New Guinea nearly all cases of tonal languages have long or intermediately long words.

## Discussion

This paper has demonstrated the existence of a relationship between the number of tonal distinctions and mean word length. When controlling for membership in different world areas and language families, this relationship remains highly significant. The finding from linear mixed effect modeling that around half a tonal distinction is gained per one segment decrease of word length suggests that the relationship, apart from being significant, is also relatively strong. We did note, however, that the prediction from word length seems to break down beyond three tonal distinctions—the number of tones that a complex system reckons with may largely be unrelated to mean word length, presumably because the limit to how short words can be on average (2–3 segments) is reached before the limit to how many tonal distinctions a language can develop. An example of a language where tonal contrasts initially developed through segmental loss and subsequently through other means is Vietnamese. According to Haudricourt (1954) a system of three tones, originally developed through segmental loss, further developed into a system of six tones through a merger of initial voiced and voiceless consonants. In general, developments of complex tone systems through the loss of a voicing distinction are common (e.g., Pittayaporn and Kirby, 2017 on the Tai dialect of Cao Bang and Ferlus, 2009 on Chinese with further references and general discussion).

The phylogenetic correlation analysis confirmed the existence of coupled evolution of word length and tone in many language families pertaining to the following major world macroareas: Eurasia (Austroasiatic, Indo-European), Africa (Atlantic-Congo, Afro-Asiatic, Ta-Ne-Omotic, Central Sudanic), and America (Athabaskan-Eyak-Tlingit, Otomanguean). It would be a great oversimplification to only attempt to explain the evolution of tonal systems through the loss of segmental material, though. This is not the only pathway to tones (cf. examples given in the previous paragraph and Michaud and Sands, 2020 for a recent overview). Moreover, it is also possible to imagine that the introduction of a tonal system could precede a loss of segments. Still, the relationship identified makes good sense in the light of a causal mechanism where a frequent initial motivation for the presence of tones would be to compensate for the lack of expressive materials as lexical morphemes become shorter. Earlier work (Wichmann et al., 2011; Wichmann and Holman, 2023) has demonstrated a negative correlation between word length and (log) population sizes. Taken together, the findings suggest a causal chain where larger populations lead to shorter words through general complexity reduction, and tonal systems subsequently emerge and spread among languages in order to maintain lexical distinctions, compensating for the loss of expressive means.

Mapping the geographical distribution of tonal languages with short vs. intermediate vs. long words suggests that the causal relationship is most prominent in Subsaharan Africa and SE Asia, two areas associated with Neolithic revolutions and large prehistorical population booms (Bellwood, 2004). Thus, short words and tone tends to be an areally concentrated ‘package’ which is furthermore often associated with large populations probably ultimately related to the impact of agriculture. This suggests that it would have been much less frequent among the world’s languages in pre-Neolithic times than nowadays. Exploring the implications of the relationship between word length and tones for the prehistory of languages and their speakers requires more work and is a fascinating item for future research.

## Data availability statement

The datasets presented in this study can be found in online repositories. The names of the repository/repositories and accession number(s) can be found at: https://github.com/Sokiwi/Tone-WordLength, https://zenodo.org/record/6344024.

## Author contributions

SW conceived and designed the study, prepared the data, performed the statistical analysis, and wrote the manuscript.

## Funding

This work was supported by the Deutsche Forschungsgemeinschaft (German Research Foundation) under Germany’s Excellence Strategy (grant EXC 2150 390870439).

## Conflict of interest

The author declares that the research was conducted in the absence of any commercial or financial relationships that could be construed as a potential conflict of interest.

## Publisher’s note

All claims expressed in this article are solely those of the authors and do not necessarily represent those of their affiliated organizations, or those of the publisher, the editors and the reviewers. Any product that may be evaluated in this article, or claim that may be made by its manufacturer, is not guaranteed or endorsed by the publisher.

## Footnotes

^1^ https://wals.info/languoid/samples/100

^2^ https://phoible.org/contributors/PH

^3^https://evolution.genetics.washington.edu/phylip/newicktree.html, for instance.

## References

[ref1] Bates D. Maechler M. Bolker B. Walker S. (2015). Fitting linear mixed-effects models using lme4. J. Stat. Softw. 67, 1–48. doi: 10.18637/jss.v067.i01

[ref2] Bellwood P. (2004). First farmers: the origins of agricultural societies. Malden, MA: Blackwell Publishing.

[ref3] Bennett R. (2016). Mayan phonology. Lang. Linguist. Compass 10, 469–514. doi: 10.1111/lnc3.12148

[ref4] Bickel B. Nichols J. Zakharko T. Witzlack-Makarevich A. Hildebrandt K. Rießler M.. (2017). The AUTOTYP typological databases. Version 0.1.0. Available at: https://zenodo.org/record/3667562#.YineCJYo9EY

[ref5] Brown C. H. Holman E. W. Wichmann S. (2013). Sound correspondences in the world’s languages. Language 89, 4–29. doi: 10.1353/lan.2013.0009, PMID: 35217676

[ref6] Dediu D. (2018). Making genealogical language classifications available for phylogenetic analysis: Newick trees, unified identifiers, and branch length. Lang. Dyn. Chang. 8, 1–21. doi: 10.1163/22105832-00801001

[ref7] Dellert J. Daneyko T. Münch A. Ladygina A. Buch A. Clarius N.. (2020). NorthEuraLex: a wide-coverage lexical database of Northern Eurasia. Lang. Resources Eval. 54, 273–301. doi: 10.1007/s10579-019-09480-6, PMID: 32214931 PMC7067722

[ref8] Dray S. Dufour A. (2007). The ade4 package: implementing the duality diagram for ecologists. J. Stat. Softw. 22, 1–20. doi: 10.18637/jss.v022.i04

[ref9] Dryer M. S. Haspelmath M. Eds. (2013). The world atlas of language structures online. (Leipzig: Max Planck Institute for Evolutionary Anthropology).

[ref10] Ferlus M. (2009). What were the four divisions of middle Chinese? Diachronica 26, 184–213. doi: 10.1075/dia.26.2.02fer, PMID: 36971353

[ref11] Hammarström H. Forkel R. Haspelmath M. Bank S. (2021). Glottolog 4.4. Leipzig: Max Planck Institute for Evolutionary Anthropology.

[ref12] Harvey P. H. Pagel M. D. (1991). The comparative method in evolutionary biology. (Oxford: Oxford University Press).

[ref13] Haudricourt A.-H. (1954). De l’origine des tons en vietnamien. J. Asiat. 242, 69–82.

[ref14] Holman E. W. Wichmann S. Brown C. H. Velupillai V. Müller A. Bakker D. (2008). “Advances in automated language classification” in Quantitative investigations in theoretical linguistics. eds. Arppe A. Sinnemäki K. Nikanne U. (Helsinki: University of Helsinki), 40–43.

[ref15] Jäger G. (2018). Global-scale phylogenetics linguistic inference from lexical resources. Sci. Data 5:180189. doi: 10.1038/sdata.2018.189, PMID: 30299438 PMC6176785

[ref16] Maddieson I. (2007). “Issues of phonological complexity: statistical analysis of the relationship between syllable structures, segment inventories, and tone contrasts” in Experimental approaches to phonology. eds. Solé M.-J. Beddor P. S. Ohala M. (Oxford: Oxford University Press), 93–103.

[ref17] Maddieson I. (2013). “Tone” in The world atlas of language structures online. eds. Dryer M. S. Haspelmath M. (Leipzig: Max Planck Institute for Evolutionary Anthropology)

[ref18] Meade A. Pagel M. (2023). BayesTraits V4.0.1. Software and manual. Available at: http://www.evolution.reading.ac.uk/BayesTraitsV4.0.1/BayesTraitsV4.0.1.html.

[ref19] Michaud A. Sands B. (2020). “Tonogenesis” in Oxford research Encyclopedia of linguistics. ed. Aronoff M. (Oxford: Oxford University Press)

[ref20] Moran S. P. (2012). Phonetics information base and lexicon. Ph.D. Dissertation. Seattle, WA: University of Washington.

[ref21] Moran S. Bentz C. Gutierrez-Vasques X. Sozinova O. Samardzic T. (2022). TeDDi sample: text data diversity sample for language comparison and multilingual NLP. Proceedings of the 13th Conference on Language Resources and Evaluation (LREC 2022), Marseille, 20–25 June 2022, 1150–1158.

[ref22] Moran S. Blasi D. (2014). “Cross-linguistic comparison of complexity measures in phonological systems” in Measuring grammatical complexity. eds. Newmeyer F. J. Preston L. B. (Oxford: Oxford University Press), 217–240.

[ref23] Moran S. McCloy D. (Eds). (2019). PHOIBLE 2.0. Jena: Max Planck Institute for the Science of Human History.

[ref24] Müller K. Wickham H. (2022). tibble: Simple Data Frames. R package version 3.1.8. Available at: https://CRAN.R-project.org/package=tibble.

[ref25] Nettle D. (1995). Segmental inventory size, word length, and communicative efficiency. Linguistics 33, 359–367.

[ref26] Nettle D. (1998). Coevolution of phonology and the lexicon in twelve languages of West Africa. J. Quant. Linguist. 5, 240–245. doi: 10.1080/09296179808590132

[ref27] Nikolaev D. (2018). The database of Eurasian phonological inventories: a research tool for distributional phonological typology. Linguist. Vanguard 4:20170050. doi: 10.1515/lingvan-2017-0050

[ref28] Pagel M. (1994). Detecting correlated evolution on phylogenies: a general method for the comparative analysis of discrete characters. Proc. R. Soc. Lond. B 255, 37–45. doi: 10.1098/rspb.1994.0006

[ref29] Pagel M. (1997). Inferring evolutionary processes from phylogenies. Zool. Scripta 26, 331–348. doi: 10.1111/j.1463-6409.1997.tb00423.x, PMID: 37097343

[ref30] Pagel M. (1999). Inferring the historical patterns of biological evolution. Nature 401, 877–884. doi: 10.1038/44766, PMID: 10553904

[ref31] Pagel M. Meade A. Barker D. (2004). Bayesian estimation of ancestral character states on phylogenies. Syst. Biol. 53, 673–684. doi: 10.1080/10635150490522232, PMID: 15545248

[ref32] Paradis E. Schliep K. (2019). Ape 5.0: an environment for modern phylogenetics and evolutionary analyses in R. Bioinformatics 35, 526–528. doi: 10.1093/bioinformatics/bty633, PMID: 30016406

[ref33] Pittayaporn P. Kirby J. (2017). Laryngeal contrasts in the Tai dialect of Cao Bằng. J. Int. Phon. Assoc. 47, 65–85. doi: 10.1017/S0025100316000293

[ref34] R Core Team (2022). R: A language and environment for statistical computing. (Vienna, Austria: R Foundation for Statistical Computing).

[ref35] Raftery A. E. (1996). “Hypothesis testing and model selection” in Markov chain Monte Carlo in practice. eds. Gilks W. R. Richardson S. Spiegelhalter D. J. (Dordrecht: Springer Science + Business Media), 163–187.

[ref36] Revell L. (2011). For fun: least squares phylogeny estimation. Blog post. Available at: http://blog.phytools.org/2011/03/for-fun-least-squares-phylogeny.html

[ref37] Round E. R. (2021). glottoTrees: phylogenetic trees in linguistics. R package version 0.1. Available at: https://github.com/erichround/glottoTrees.

[ref38] Sagart L. (1999). “The origin of Chinese tones” in Cross-linguistic studies of tonal phenomena: tonogenesis, typology, and related topics. ed. Kaji S. (Tokyo: ILCAA), 91–103.

[ref39] Schliep K. P. (2011). phangorn: phylogenetic analysis in R. Bioinformatics 27, 592–593. doi: 10.1093/bioinformatics/btq706, PMID: 21169378 PMC3035803

[ref40] Shcherbakova O. Gast V. Blasi D. E. Skirgård H. Gray R. D. Greenhill S. J. (2022). A quantitative global test of the complexity trade-off hypothesis: the case of nominal and verbal grammatical marking. Linguistics Vanguard. doi: 10.1515/lingvan-2021-0011

[ref41] Simons G. F. Fennig C. D. (Eds.) (2017). Ethnologue: languages of the world, 20th. (Dallas, TX: SIL International).

[ref42] South A. (2011). rworldmap: a new R package for mapping global data. R. J. 3, 35–43. doi: 10.32614/RJ-2011-006

[ref43] Tamura K. Stecher G. Kumar S. (2021). MEGA11: molecular evolutionary genetics analysis version 11. Mol. Biol. Evol. 38, 3022–3027. doi: 10.1093/molbev/msab120, PMID: 33892491 PMC8233496

[ref44] Wichmann S. (2013). “A classification of Papuan languages” in History, contact and classification of Papuan languages language and linguistics in Melanesia, Special Issue 2012. eds. Hammarström H. van den Heuvel W. (Port Moresby: Linguistic Society of Papua New Guinea), 313–386.

[ref45] Wichmann S. (2023). InteractiveASJP02. Software available at: https://github.com/Sokiwi/InteractiveASJP02.

[ref46] Wichmann S. Holman E. W. (2023). Cross-linguistic conditions on word length. PLoS One 18:e0281041. doi: 10.1371/journal.pone.0281041, PMID: 36706125 PMC9882889

[ref47] Wichmann S. Holman E. W. Bakker D. Brown C. H. (2010a). Evaluating linguistic distance measures. Physica A 389, 3632–3639. doi: 10.1016/j.physa.2010.05.011

[ref70] Wichmann S. Holman E. W. Brown C. H. (Eds.) (2022) The ASJP database (version 20). Available at: https://asjp.clld.org/.

[ref48] Wichmann S. Holman E. W. Brown C. H. (Eds.) (2020) The ASJP database (version 19). Available at: https://asjp.clld.org/.

[ref49] Wichmann S. Müller A. Velupillai V. (2010b). Homelands of the world’s language families: a quantitative approach. Diachronica 27, 247–276. doi: 10.1075/dia.27.2.05wic

[ref50] Wichmann S. Rama T. Holman E. W. (2011). Phonological diversity, word length, and population sizes across languages: the ASJP evidence. Linguist. Typol. 15, 177–197. doi: 10.1515/LITY.2011.013

[ref51] Wickham H. (2016). ggplot2: elegant graphics for data analysis. (New York: Springer-Verlag).

[ref52] Wickham H. (2022). stringr: simple, consistent wrappers for common string operations. R package version 1.5.0. Available at: https://CRAN.R-project.org/package=stringr.

[ref53] Wickham H François R., Henry L., Müller K., and Vaughan D. (2023). dplyr: a grammar of data manipulation. R package version 1.1.0. Available at: https://CRAN.R-project.org/package=dplyr.

[ref54] Zeileis A. Fisher J. C. Hornik K. Ihaka R. McWhite C. D. Murrell P.. (2020). colorspace: a toolbox for manipulating and assessing colors and palettes. J. Stat. Softw. 96, 1–49. doi: 10.18637/jss.v096.i01

